# Students’ continuance intention to use MOOCs: empirical evidence from India

**DOI:** 10.1007/s10639-022-11308-w

**Published:** 2022-10-14

**Authors:** I S Rekha, Jyothi Shetty, Savitha Basri

**Affiliations:** 1 Manipal Institute of Management, Manipal Academy of Higher Education, Manipal, Karnataka India; 2 Department of Commerce, Manipal Academy of Higher Education, Manipal, Karnataka India

**Keywords:** Computer self-efficacy, Confirmation, Enjoyment, MOOCs, Openness, Perceived usefulness, Reputation, Satisfaction

## Abstract

In recent years, there has been an increasing interest in understanding the Massive open online courses (MOOCs) due to its gaining popularity. Even though the number of online platforms and programs has grown during the COVID-19 pandemic, there is still a high rate of dropout and non-completion. In this work, the expectation-confirmation model is combined with MOOC features such as perceived openness, perceived reputation, and other factors i.e., perceived enjoyment, and perceived computer self-efficacy to investigate the learner’s continued intention to use MOOC. A survey was undertaken and the data was collected from 383 students pursuing their degrees (undergraduate and post-graduate) in Karnataka state, India. The collected data were analyzed with structural equation modelling in Smart PLS 3. The study confirms a significant influence of confirmation and perceived usefulness on satisfaction, and direct significant influence of perceived computer self-efficacy, satisfaction, and perceived usefulness on continuance intention. Also, the results demonstrated the significant influence of confirmation on perceived enjoyment and usefulness and the effect of computer self-efficacy on usefulness. The findings in this study indicate that the MOOC platforms should focus on confirming learner expectations and the usefulness of courses to ensure student satisfaction and continuance of courses.

## Introduction

In recent times, Information and Communication Technology has boosted learning to greater heights and digitalization has made this possible for learners to move into the digital e-learning era (Wu, 2021). MOOCs are the courses offered via the internet or online mode to a large number of learners for free of charge or nominal fees. The courses usually include short video lectures, reading materials, quizzes, and web exercises. Due to the pandemic in the year 2020, MOOCs had reached over 180 million learners with about 950 universities offering various courses (Shah, 2020). Udacity, edX, Coursera, and Swayam are some of the known MOOC platforms. A learner may enjoy the benefits of MOOC learning like flexibility, accessibility, and cost-effectiveness, however, one of the challenges faced by these providers is the high dropout rate or low completion rate (Cagiltay et al., 2020; Bartolome & Steffens, 2015; Jordan, 2014; Li et al., 2018; Macleod et al., 2015). On average, less than 10% of learners enrolled in MOOCs would complete the enrolled course (Wu & Chen, 2017; Alraimi et al., 2015; Zhang et al., 2018; Xing et al., 2016). The completion rate has remained low over time, with no signs of improvement (Sun et al., 2020). There can be numerous reasons for drop-out such as no real intention to complete, importance to learning experience rather than completion, course difficulty, and different expectations (Onah et al., 2014; Chang et al., 2015). Because educational gains are dependent on course completion and the viability and sustainability of MOOCs are determined by learner continuance intention (Bhattacherjee & Premkumar, 2004; Barnes, 2011; Wan et al., 2020), a study of factors influencing continuance intention and not short-term usage is required to understand the ultimate success of MOOCs (Daghan & Akkoyunlu, 2016; Nikou & Economides, 2017; Chao, 2019). Although, there have been studies utilizing the survey analysis and case study technique to investigate MOOC retention (Greene et al., 2015; DeFreitas et al., 2015), very less is learned about the intentions of MOOC learners to continue taking MOOCs during this pandemic, when students experienced problematic internet use and academic burnout, resulting in impaired learning (Basri et al., 2022).

During this pandemic of COVID-19, when most of the higher education institutions in several countries adopted emergency remote teaching-learning methods (Al-Kumaim et al., 2021) and incorporated MOOCs as an open elective, certain intrinsic characteristics of the platform became relevant for not only selecting the courses but also its continuance intention to use. Because of the huge number of courses offered on MOOC platforms during the COVID-19 epidemic, students’ willingness to complete the courses is determined by the perceived usefulness of courses, confirmation of prior expectations, and satisfaction with the learning experience (expectation-confirmation model), MOOC features as well as learner characteristics. Any student contemplating completing a course on a MOOC’s platform is likely to be concerned with the platform’s reputation, enjoyment, happiness, and openness, all of which are essential features of MOOCs (Alraimi et al., 2015).

Chen et al., (2018) define openness as open communication and resource sharing and transparent curriculum structure and objectives. It is the degree of freedom that users relish in accessing and using course resources at free of cost such as presentation slides, and course videos, without any enrollment prerequisites (Alraimi et al., 2015). The perceived reputation of the institution offering the course affects the enrollment and continuance decision. The reputation of a higher education institution is defined as its image in the eyes of others in terms of quality, influence, and trustworthiness (Van Vught, 2008). It denotes the institution’s perceived greatness, which influences the decision to join based on a preliminary evaluation (Wu & Chen, 2017). Therefore, this study tries to address MOOC features i.e., perceived openness and perceived enjoyment (Alraimi et al., 2015) in understanding satisfaction.

When learning takes place on an online platform, it is critical that the learner be intrinsically motivated to participate in the learning and that the learner be able to finish his/her work using a computer. Davis et al., (1992) investigated perceived enjoyment on usage intention. Enjoyment is the extent to which using technology is perceived to be entertaining, which is apart from any anticipated performance consequences. Indeed, enjoyment plays an important part in the successful performance of a technology (Alyoussef, 2021). The levels of motivation among people are determined by their self-efficacy beliefs, which are reflected in the amount of effort they put in and how long they will persevere in the face of an obstacle. If the belief in their capability is stronger, then the efforts are greater and are more persistent (Bandura, 1988, 1989; Rabaa’i et al., 2021). Computer self-efficacy can be defined as the ability of a person to perform tasks using a computer (Shahbaz et al., 2020). Therefore, this study tries to address the influence of other features relevant to MOOC learning namely perceived enjoyment (Jimenez et al., 2021; Tao et al., 2019) and computer self-efficacy (Al-Adwan, 2020; Jimenez et al., 2021) in context of MOOCs continuous use during the COVID-19 pandemic.

Most behavioural theories measure the adoption intention of users whereas expectation-confirmation theory (ECT) studies both variables related to the pre-consumption and the post-consumption. The expectation-confirmation model (ECM), as an improvised version of ECT, focuses mainly on post-acceptance factors because the effect of pre-acceptance factors is already captured in confirmation and satisfaction (Bhattacherjee, 2001a). According to Bhattacherjee (2001a), continuance intention is the intention of the user to continue the usage of the system. Consequently, the current study is an extension of ECM (Bhattacherjee, 2001a) that incorporates MOOC’s features namely perceived openness and perceived reputation (Alraimi et al., 2015) and other significant variables (perceived enjoyment and computer self-efficacy). Therefore, the objective is to examine the antecedents to satisfaction and continuance intention to use MOOCs by students, and also to examine the impact of perceived computer self-efficacy on perceived usefulness and the influence of confirmation on perceived enjoyment and perceived usefulness.

### Conceptual model

#### Expectation confirmation theory (ECT) and expectation confirmation model (ECM)

ECT conceptualized by Oliver (1980) views post-purchase satisfaction as an outcome of user expectation, the perceived performance (regarding a service or a product), and disconfirmation of beliefs. While Bhattacherjee (2001a) proposes a post-acceptance model of information system (IS) continuance intention, it focuses primarily on post-adoption determinants, as the pre-adoption components are covered under confirmation and satisfaction. As the expectation may change with experience, the model studies ex-post expectation which is termed perceived usefulness. ECT inspects both the pre-consumption and the post-consumption factors whereas the ECM, as an improvised version of ECT, focuses mainly on post-acceptance factors (Bhattacherjee, 2001a). The ECM, a post-acceptance model (Bhattacherjee, 2001a), is most suited for this study because the main aim of this study is to recognize the factors that influence students’ desire to continue using MOOCs.

**Fig. 1:**
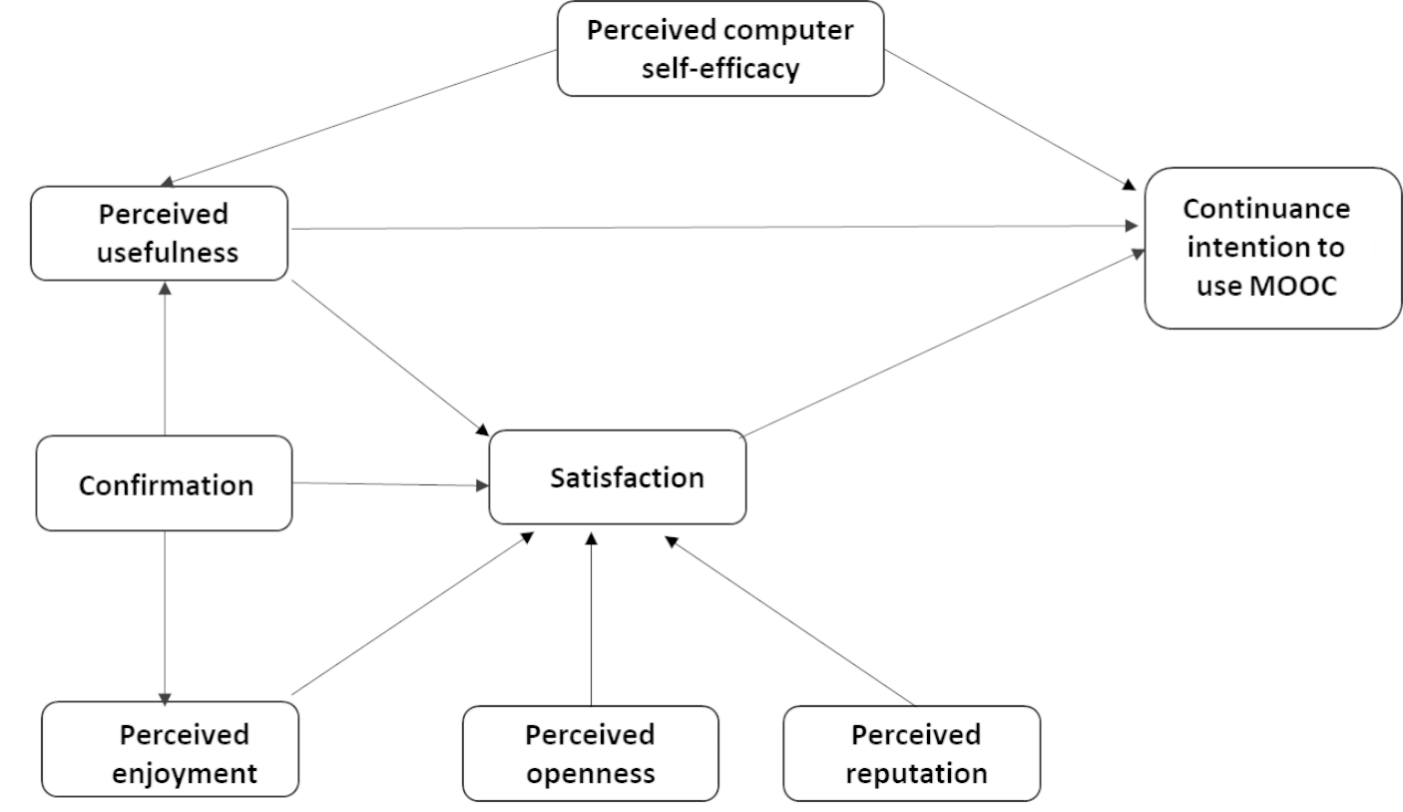
Conceptual Model

Bhattacherjee’s (2001a) ECM model is based on five hypotheses. Derived from ECT and evidence from prior studies, ECM hypothesizes that the satisfaction from initial use positively affects continuance intention to use technology/IS. The ECM confirms the positive association between confirmation and satisfaction since confirmation implies that the user’s expectations of IS use are met. It asserts that users’ concerns about ease of use are alleviated as they gain familiarity with the system, and therefore, it suggests that perceived usefulness is the ex-post expectation influencing satisfaction, which is a post-acceptance effect. Built on the technology acceptance model (TAM) that hypothesizes the positive association between perceived usefulness and intention, ECM tests the association in the continuance intention context established on the assumption that the behaviour is independent of timing or stage of behaviour. It states that the confirmation of past expectations will increase the user’s perceived usefulness and vice versa. Since the ECM model has been thoroughly covered in the literature (Bhattacherjee, 2001a, b; Bhattacherjee & Premkumar, 2004; Dai et al., 2020; Ouyang et al., 2017; Yang et al., 2022; Rahmania et al., 2022; Suriazdin et al., 2022; Nong et al., 2022; Lee, 2010; Gupta et al., 2020; Malik & Rao, 2019; Jin et al., 2010), the current study just briefly summarises the theory. The conceptual model (Fig. 1), which is based on the ECM model (Bhattacherjee, 2001a) and other previous investigations, identifies initial results that may be investigated further.

## Hypotheses development

### Expectation confirmation model

Several studies have applied ECM in its original form or extended version to study the continuance intention of users of various technological innovations. For example, while studying continuance intention of usage of MOOC among university students, Dai et al., (2020) could not establish the direct influence of confirmation on continuance intention to use MOOC but proved its influence on satisfaction. Similarly, Rahmania et al., (2022) studies students’ continuance intention towards e-learning and they investigated all hypotheses of ECM and could establish significant results in case of all except the influence of perceived usefulness on satisfaction. However, Yang et al., (2022) examined the continuance intention of beginners in higher education in blended learning and found significant result for all hypotheses of ECM. Lee (2010) continued with ECM to study continuance intention in e-learning and the findings confirmed the significant influence of satisfaction, perceived usefulness, concentration, subjective norm, attitude, and perceived behavioural control on continuance intention. The author also reported a significant impact of confirmation on perceived usefulness and satisfaction. Ouyang et al., (2017) combined the task technology fit (TTF) model with the ECM and observed the direct effect of perceived usefulness, satisfaction, and TTF on continuance intention to use MOOC, and also confirmation on both perceived usefulness and satisfaction. Thus, rather than examining ECM as a stand-alone model, including pertinent MOOC components might improve our knowledge of continuance intention. Such attempts were made by Suriazdin et al., (2022) who combined ECM with technology attractiveness and they discovered findings that were in line with those of ECM, with the exception of the perceived usefulness on continuing intention. Nong et al., (2022) combined ECM with perceived service quality in examining the MOOC continuance intention in China and the results corroborated the ECM findings and in addition found the significant influence of perceived service quality on satisfaction and continuation intention. In other contexts, Gupta et al., (2020) combined UTAUT (pre-adoption performance expectancy and effort expectancy) and post-adoption ECM (along with perceived usefulness and satisfaction, the other post-adoption factors are user interface quality, self-efficacy, security) in continuance intention to use M-wallets, Malik & Rao (2019) applied extended ECM in predicting the continuance intention of users of on-demand ride services.Jin et al., (2010) extended ECM in understanding continuance intention of users to take part in an online community. Therefore, based on ECM and evidence from prior studies, we derived the following hypotheses:

H1: MOOC users’ level of confirmation is positively associated with satisfaction derived from MOOC use.

H2: Users’ perceived usefulness of MOOC is positively associated with satisfaction derived from MOOC use.

H3: MOOC users’ level of confirmation is positively associated with perceived usefulness of MOOC use.

H4: Perceived usefulness has a positive impact on continuance intention to use MOOC.

H5: MOOC users’ level of satisfaction is positively associated with the continuance intention to use MOOC.

### Variables in the context of MOOC

In addition to ECM variables, we propose to include MOOC features namely perceived opennes and perceived reputation, and other learner-specific antecedents such as perceived enjoyment and computer self-efficacy.

#### Perceived openness

Although open communication, resource sharing, and openness in curriculum structure and goals are crucial, there is relatively little research in this area. Openness will provide easy access to course resources which will enhance satisfaction (Chen et al., 2018; Choi et al., 2019) as well as perceived usefulness (Choi et al., 2019). Even Khurana et al., (2019) confirmed the positive influence of openness on satisfaction. Therefore, we hypothesize that,

H6: Perceived openness has a positive impact on satisfaction.

#### Perceived reputation

The perceived reputation of the institution, which signals its eminence, affects the decision to enrol (Wu & Chen, 2017). The most popular MOOC platforms are affiliated with prestigious schools, and as a result, they are likely to gain rapid credibility. Shahijan et al., (2016) established that a university’s reputation positively influences course satisfaction. Alraimi et al., (2015) considered perceived reputation as the inherent feature of MOOC and confirmed a direct relationship between reputation and satisfaction.

Therefore, we hypothesize that,

H7: Perceived reputation has a positive impact on satisfaction.

#### Perceived enjoyment

Belief in MOOC enjoyment is a hedonic motivating factor to use the latest technology. Due to the ability to study at their own speed and increased sense of enjoyment, users are more likely to find MOOCs to be interesting (Tao et al., 2019). If the users perceive that the use of MOOC is enjoyable then it is more likely to have favourable feelings regarding perceived ease of use (PEOU), perceived usefulness, and intention to use. Oghuma et al., (2016) state that if the technology/system use carries an enjoyment factor, it will not just influence the intention or continuance intention to use but also the satisfaction from use. Kim (2011); Alraimi et al., (2015); Oghuma et al., (2016); Abdullahi et al., (2021) found a significant influence of confirmation on perceived enjoyment. Alraimi et al., (2015); Oghuma et al., (2016); Chao (2019); Huang (2020) and Abdullahi et al., (2021) reported a direct influence of perceived enjoyment on satisfaction.

Accordingly, the following two hypotheses were formulated:

H8: Perceived enjoyment in using MOOC has a positive impact on satisfaction.

H9: Confirmation of expectation has a positive impact on perceived enjoyment.

#### Perceived computer self-efficacy

Compeau & Higgins (1995) state that people having higher self-efficacy are unlikely to be frustrated or discouraged by barriers and are more likely to get over them owing to their perseverance. An individual with higher computer self-efficacy is likely to have higher perceived usefulness, ease of use, and willingness to utilize it. A person with a higher computer self-efficacy perceives to be capable of handling computer-related work with less support and would be competent to use different systems (Wangpipatwong et al., 2008). Numerous studies have confirmed the significant influence of computer self-efficacy on intention/continuance intention to use the system (Wangpipatwong et al., 2008; Chiu & Wang, 2008; Alenezi & Karim, 2010; Lew et al., 2019; Fianu et al., 2018). It is stated that (Al-Rahmi et al., 2020) if students perceive that they can use computers, then they will be inclined to use an ICT that permits them to be productive in fulfilling their tasks i.e., higher perceived computer self-efficacy leads to higher perceived usefulness. More than a few studies that have investigated the influence of computer self-efficacy on perceived usefulness (Jiang et al., 2021; Sayaf et al., 2021; Al-Rahmi et al., 2020; Al-Adwan, 2020; John, 2013) found a direct influence of computer self-efficacy on perceived usefulness.

Therefore, we hypothesize that,

H10: Perceived computer self-efficacy has a significant impact on perceived usefulness.

H11: Perceived computer self-efficacy has a significant impact on continuance intention to use MOOC.

The conceptual model is shown in Fig. 1. We propose three antecedents to continuance intention to use MOOC (perceived usefulness, satisfaction, perceived computer self-efficacy), the direct positive influence of five constructs on satisfaction (perceived usefulness, confirmation, perceived openness, perceived enjoyment, perceived reputation), and the influence of perceived computer self-efficacy on perceived usefulness; and confirmation on perceived enjoyment and perceived usefulness concerning MOOC.

## Research methodology

The research falls under the positivist paradigm of research as it believes that the information/elements involved in this study are measurable facts, and is data-driven. Therefore, the research approach is based on a quantitative with a single method i.e, the survey method of research inquiry. The units of observation in this study are individuals who have experience in MOOC learning. It is a cross-sectional analytical study that empirically tested the study hypotheses. The data analysis was done using structural equation modelling with Smart PLS ver 3.

### Survey instrument

The study uses the structured questionnaire as the data collection instrument to collect the required data. There were two sections in the survey instrument. 33 statements were used to measure eight constructs of the research model. Agreeableness on the Likert scale of 1 to 5 was used to measure all the constructs involved in the conceptual model, wherein 5 = strongly agree and 1 = strongly disagree. All the measures of each construct were based on past studies and the statements were slightly modified. Four items of perceived usefulness were taken from Venkatesh et al., 2012. Items measuring continuance intention and satisfaction were adapted from Bhattacherjee (2001b) and confirmation was adapted from Alraimi et al., (2015). Perceived enjoyment, perceived openness, perceived reputation, and perceived computer self-efficacy were adapted from Lee (2010); Alraimi et al., (2015); Kim (2008) & Alraimi et al., (2015); and Roca et al., (2006) respectively.

### Sampling process

Snowball sampling technique was chosen due to the nationwide lockdown during the COVID-19 pandemic in India during which all the colleges were functioning in online mode, and hence the size of the population and sampling frame was not available. The respondents could not be personally approached due to the same reason. Moreover, snowball sampling is known to have been used to contact difficult to reach population, and forwarding the questionnaire to someone else is an effective way of increasing the response rate (Etter & Perneger, 2000). The data was collected from a pool of undergraduate and post-graduate students who had experience in MOOC learning. The Google form link was first shared with students of selected institutes in Karnataka due to the non-availability of a sampling frame for all the states in India. Initially, a few undergraduate or postgraduate students from each institute were chosen, and they were then encouraged to recommend suitable respondents to whom questionnaires were sent in the following phase. The process was repeated until the necessary responses were gathered. Online services such as WhatsApp, emails, and social media were used to distribute the survey instrument using the Google form. The data collection took place between February-May 2020. The survey received 392 responses and only 383 responses were used for analysis. In the final sample, 49% of the respondents were female, 83% were undergraduate students and 89% of the respondents had either enrolled or completed two or more MOOCs.

## Results

### Evaluation of the measurement model

The reflective measurement model assessment was done in three steps i.e., through internal consistency reliability, convergent validity, and discriminant validity. Both composite reliability and Cronbach’s alpha had a loading of greater than 0.7 (thereby, confirming that the all the items measuring a construct are similar in their scores, Hair et al., 2017). Convergent validity measures the extent to which a measure correlates positively with other measures of the same construct (Hair et al., 2017). The convergent validity was established through outer loading and average variance extracted (AVE). The four items related to continuance intention and satisfaction (CI3, SAT3, SAT4, and SAT6 (Refer Table 1)) not meeting the criteria were eliminated from further analysis and the remaining measures carry an outer loading of greater than 0.708 (Hair et al., 2017) (Table 1). The convergent validity at the level of the construct was evaluated through AVE and the minimum threshold was 0.5. All the constructs have an AVE of more than 0.8 and perceived openness had an AVE of 0.696 (Table 1), thereby all the constructs meet the convergent validity. To evaluate if the constructs in the study were truly distinct from each other, the discriminant validity was assessed through both traditional criteria (cross-loading and Fornell-Larcker Criterion) and the new criteria HTMT (Heterotrait-Monotrait Ratio) (Table 2). The cross-loading and Fornell-Larcker Criterion established discriminant validity. The discriminant validity through HTMT could not be established for the two items of perceived usefulness i.e., PU1 (consistent with Gefen 2003) and PU2 (consistent with Venkatesh et al., 2012) which were strongly correlated with other constructs and hence were eliminated from further analysis.

**Table 1:** Indicator and construct reliability and validity

Construct	Indicators	Outer Loading	Cronbach’s Alpha	Composite Reliability	Average Variance Extracted (AVE)
CI	CI1 CI2	0.932 0.947	0.867	0.937	0.882
CONF	CONF1 CONF2 CONF3	0.935 0.940 0.928	0.927	0.954	0.873
PCSE	PCSE1 PCSE2 PCSE3 PCSE4	0.845 0.916 0.910 0.915	0.919	0.943	0.805
PENJ	PENJ1 PENJ2 PENJ3	0.952 0.953 0.954	0.949	0.967	0.908
PO	PO1 PO2 PO3 PO4 PO5 PO6	0.859 0.841 0.885 0.708 0.874 0.827	0.914	0.932	0.696
PREP	PREP1 PREP2 PREP3 PREP4	0.929 0.936 0.918 0.904	0.941	0.958	0.850
PU	PU3 PU4	0.955 0.954	0.902	0.953	0.911
SAT	SAT1 SAT2 SAT5	0.933 0.934 0.885	0.906	0.941	0.843

Note: CI: continuance intention; CONF: confirmation; PCSE: perceived computer self-efficacy; PENJ: perceived enjoyment; PO: perceived openness; PREP: perceived reputation; PU: perceived usefulness; SAT: satisfaction

**Table 2:** Discriminant validity

	CI	CONF		PCSE	PENJ	PO	PREP	PU		SAT
**a. Fornell-Larcker Criterion**										
CI	**0.939**									
CONF	0.729	**0.935**								
PCSE	0.645	0.669		**0.897**						
PENJ	0.700	0.746		0.708	**0.953**					
PO	0.608	0.597		0.713	0.655	**0.835**				
PREP	0.709	0.745		0.745	0.799	0.690	**0.922**			
PU	0.760	0.816		0.634	0.759	0.596	0.747	**0.955**		
SAT	0.638	0.730		0.520	0.642	0.527	0.636	0.683		**0.918**
**b. Cross Loading**										
CI1	**0.932**		0.646	0.556	0.595	0.555	0.626	0.673		0.567
CI2	**0.947**		0.721	0.651	0.714	0.585	0.702	0.750		0.628
CON1	0.672		**0.935**	0.600	0.709	0.559	0.674	0.741		0.692
CON2	0.651		**0.940**	0.621	0.686	0.558	0.694	0.775		0.667
CON3	0.722		**0.928**	0.656	0.697	0.556	0.720	0.771		0.686
PCSE1	0.603		0.551	**0.845**	0.594	0.643	0.627	0.544		0.447
PCSE2	0.571		0.588	**0.916**	0.624	0.649	0.643	0.559		0.466
PCSE3	0.570		0.634	**0.910**	0.641	0.631	0.676	0.570		0.466
PCSE4	0.570		0.627	**0.915**	0.680	0.634	0.724	0.598		0.486
PENJ1	0.674		0.717	0.668	**0.952**	0.607	0.764	0.719		0.619
PENJ2	0.682		0.708	0.669	**0.953**	0.638	0.766	0.737		0.607
PENJ3	0.645		0.708	0.687	**0.954**	0.627	0.755	0.715		0.609
PO1	0.600		0.649	0.652	0.713	**0.859**	0.703	0.619		0.567
PO2	0.447		0.414	0.541	0.456	**0.841**	0.500	0.398		0.362
PO3	0.458		0.474	0.552	0.481	**0.885**	0.548	0.456		0.424
PO4	0.322		0.273	0.417	0.338	**0.708**	0.326	0.299		0.234
PO5	0.556		0.493	0.641	0.556	**0.874**	0.597	0.530		0.453
PO6	0.559		0.549	0.684	0.599	**0.827**	0.643	0.559		0.474
PREP1	0.659		0.721	0.712	0.769	0.632	**0.929**	0.701		0.602
PREP2	0.664		0.700	0.693	0.744	0.662	**0.936**	0.716		0.599
PREP3	0.675		0.687	0.696	0.745	0.640	**0.918**	0.693		0.573
PREP4	0.617		0.636	0.643	0.688	0.611	**0.904**	0.642		0.571
PU3	0.732		0.788	0.630	0.698	0.579	0.714	**0.955**		0.653
PU4	0.718		0.769	0.579	0.752	0.559	0.712	**0.954**		0.652
SAT1	0.586		0.673	0.480	0.588	0.479	0.596	0.626		**0.933**
SAT2	0.592		0.694	0.478	0.603	0.501	0.579	0.625		**0.934**
SAT5	0.579		0.642	0.474	0.577	0.470	0.577	0.631		**0.885**
**C. Heterotrait-Monotrait Ratio (HTMT)**										
CI										
CONF	0.811									
PCSE	0.719	0.725								
PENJ	0.768	0.795		0.758						
PO	0.659	0.618		0.759	0.673					
PREP	0.783	0.797		0.800	0.845	0.713				
PU	0.856	0.891		0.695	0.821	0.628	0.810			
SAT	0.718	0.796		0.570	0.692	0.551	0.689	0.756		

Note: Bold values indicate square root of each construct’s AVE.

### Evaluation of the structural model

**Fig. 2:**
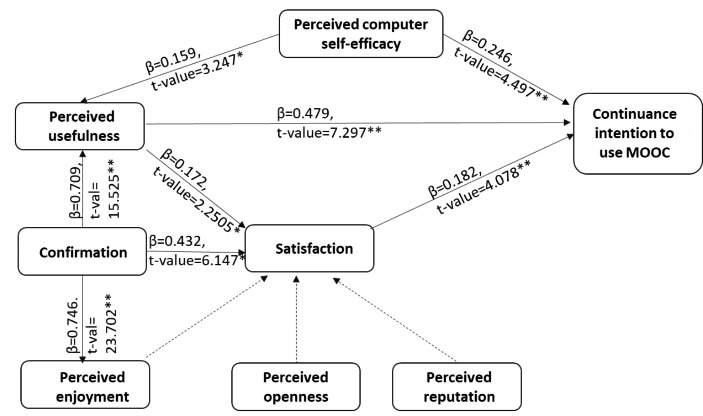
Results of the structural model. The results support 8 out of 11 hypotheses, the 3 hypotheses which are not supported are shown by dashed line indicating insignificant results (perceived enjoyment, perceived openness and perceived reputation on satisfaction)

To ensure that the predictor variables are not highly correlated and are distinct from each other, the model was first assessed for collinearity issues before checking for the significance of path coefficients. The inner variance inflation faction (VIF) values of all the predictor constructs were greater than 0.2 and lesser than 5, thereby establishing that the model is free from a collinearity issue (Table 3). The second and the most important step is to assess the significance of path coefficients i.e., assessing the significance of hypothesized relationships in the structural model. As depicted in Table 4, out of eleven hypotheses, eight were significant, but the remaining three hypotheses were rejected at a 5% significance level. Therefore, it can be concluded that (i) out of the five predictors (confirmation, perceived enjoyment, perceived openness, perceived reputation, and perceived usefulness) of satisfaction, only confirmation and perceived usefulness are significant ; (ii) all the three predictors of (perceived usefulness, satisfaction, and perceived computer self-efficacy) continuance intention are significant , (iii) perceived computer self-efficacy and confirmation significantly influence perceived usefulness, and (iv) confirmation affects perceived enjoyment (Fig. 2). The model’s predictive power was measured through the coefficient of determination (R^2^) and the values of continuance intention (63.9%), satisfaction (56.9%), perceived usefulness (67.9%), and perceived enjoyment (55.7%) can be considered to demonstrate moderate predictive power.

**Table 3:** Collinearity statistics

	CI	PENJ	PO	PREP	PU	SAT
CONF		1.000			1.812	3.548
PCSE	1.712				1.812	
PENJ						3.547
PO						2.043
PREP						3.674
PU	2.341					3.667
SAT	1.921					

**Table 4:** Structural model path analysis and explanatory power

Hypothesis	Path coefficient	Sample Mean (M)	Standard Deviation (STDEV)	t Statistics	Decision at 5% level of significance	R^2^
H1: CONF → SAT	0.432	0.435	0.070	6.147**	Supported	0.569
H8: PENJ → SAT	0.097	0.100	0.058	1.660^ns^	Not- Supported	
H6: PO → SAT	0.054	0.051	0.043	1.243^ns^	Not- Supported	
H7: PREP → SAT	0.072	0.069	0.059	1.226^ns^	Not- Supported	
H2: PU → SAT	0.172	0.170	0.069	2.505*	Supported	
H4: PU → CI	0.479	0.477	0.066	7.297**	Supported	0.639
H11: PCSE → CI	0.246	0.247	0.055	4.497**	Supported	
H5: SAT → CI	0.182	0.183	0.045	4.078**	Supported	
H3: CONF → PU	0.709	0.711	0.046	15.525**	Supported	0.679
H10: PCSE → PU	0.159	0.158	0.049	3.247	Supported	
H9: CONF → PENJ	0.746	0.745	0.031	23.702**	Supported	0.557

Note: *p < 0.05; **p < 0.01, ns: not significant

The *f*^2^ effect size indicates the change in the predictive power of the dependent variable when a predictor variable is removed from the model (Hair et al., 2017). Perceived computer self-efficacy has a small effect size of 0.043 on perceived usefulness and 0.098 on continuance intention; perceived usefulness has a medium effect size of 0.272 on continuance intention and a near to small effect size on satisfaction; satisfaction and confirmation have a small effect size of 0.048 and 0.122 on continuance intention and satisfaction respectively. confirmation has a high effect size of 0.866 on perceived usefulness and 1.257 on perceived enjoyment (Table 5). To assess the out-of-sample predictive relevance through Stone-Geisser’s (1974) Q^2^ value of the model we ran a blindfolding procedure with an omission distance (D) of seven. All the predictor variables for a dependent variable (continuance intention, satisfaction, perceived usefulness, and perceived enjoyment) had a Q^2^ value of greater than zero, thereby confirming the predictive relevance (Table 5). Further, to assess the independent variable’s contribution to the dependent variable’s Q^2^ value (Hair et al., 2017), the q^2^ effect size was calculated. The results are almost similar to that of f^2^ effect size; perceived computer self-efficacy has a small q^2^ effect size of 0.028 on perceived usefulness and 0.063 on continuance intention; perceived usefulness has a medium q^2^ effect size of 0.188 on continuance intention; confirmation has a small q^2^ effect size of 0.079 on satisfaction. On contrary to the above, confirmation has a high q^2^ effect size of 0.647 on perceived usefulness.

**Table 5:** Effect size, Predictive relevance and q2 effect size

Exogenous variable	Endogenous variable	f^2^	Q^2^ included	Q^2^ excluded	q^2^ effect size
PCSE	CI	0.098 (SE)	0.554	0.526	0.063(SE)
PU	CI	0.272 (ME)	0.554	0.470	0.188(ME)
SAT	CI	0.048 (SE)	0.554		
PU	SAT	0.019 (NE)	0.469	0.466	0.007(NE)
CONF	SAT	0.122 (SE)	0.469	0.427	0.079(SE)
PENJ	SAT	0.006 (NE)	0.469	0.468	0.003(NE)
PO	SAT	0.003 (NE)	0.469	0.470	-0.001(NE)
PREP	SAT	0.003 (NE)	0.469	0.469	0.002(NE)
PCSE	PU	0.043 (SE)	0.613	0.602	0.028(SE)
CONF	PU	0.866 (HE)	0.613	0.362	0.647(HE)
CONF	PENJ	1.257 (HE)	0.505		

Note: NE: no effect; SE: small effect HE: high effect

## Discussion

This study provides insights into the perspective of undergraduate and post-graduate students in India who were the users of MOOCs. The ECM is an established model which is empirically tested in various fields like e-learning, MOOC, and M-wallet in understanding continued intention to use the system/technology. It is not surprising to find that all the five hypotheses (confirmation → satisfaction, perceived usefulness → satisfaction, perceived usefulness → continuance intention, satisfaction → continuance intention, and confirmation → perceived usefulness) related to ECM are supported in the current study. Therefore, the findings concerning these five hypotheses are consistent with ECM and other empirical studies (Gupta et al., 2020; Malik & Rao, 2019; Ouyang et al., 2017; Lee, 2010). In addition, perceived computer self-efficacy is found to be having a direct significant impact on continuance intention and the result is consistent with that of prior studies (Wangpipatwong et al., 2008; Chiu & Wang, 2008; Lew et al., 2019). Therefore, it can be summarized that: (i) the three direct indicators of continuance intention are perceived usefulness, satisfaction, and perceived computer self-efficacy. However, confirmation (confirmation → satisfaction → continuance intention; confirmation → perceived usefulness → continuance intention; and confirmation → perceived usefulness → satisfaction → continuance intention), perceived usefulness (perceived usefulness → satisfaction → continuance intention), and perceived computer self-efficacy (perceived computer self-efficacy → perceived usefulness → continuance intention) also have a significant indirect influence on continuance intention (Refer Table 6). This has led to a total variance of 63.9% in continuance intention. (ii) perceived usefulness and confirmation are the direct indicators of satisfaction. But, confirmation also demonstrates a significant indirect effect on satisfaction through perceived usefulness (refer to Table 6) leading to total variance of 56.9%, (iii) both independent variables i.e., confirmation and self-efficacy defined as the direct indicators of perceived usefulness in the research model are significant in their relationship to perceived usefulness explaining 67.9% of the variance, and (iv) confirmation has a significant direct effect on perceived enjoyment with a variance of 55.7%.

**Table 6:** Specific indirect effect

Path	Path coefficient	Sample Mean (M)	Standard Deviation (STDEV)	t Statistics	P Values
PO → SAT → CI	0.010	0.010	0.009	1.114	0.266
**CONF → PU → CI**	**0.340**	**0.339**	**0.050**	**6.779**	**0.000**
CONF → PENJ → SAT → CI	0.013	0.013	0.008	1.565	0.118
CONF → PENJ → SAT	0.072	0.074	0.044	1.646	0.100
**CONF → PU → SAT**	**0.122**	**0.120**	**0.049**	**2.474**	**0.014**
**PCSE → PU → CI**	**0.076**	**0.076**	**0.027**	**2.764**	**0.006**
**CONF → PU → SAT → CI**	**0.022**	**0.022**	**0.010**	**2.155**	**0.032**
PCSE → PU → SAT → CI	0.005	0.005	0.003	1.800	0.072
PCSE → PU → SAT	0.027	0.027	0.014	1.947	0.052
**CONF → SAT → CI**	**0.079**	**0.080**	**0.026**	**3.028**	**0.003**
PREP → SAT → CI	0.013	0.013	0.011	1.146	0.252
PENJ → SAT → CI	0.018	0.018	0.011	1.573	0.116
**PU → SAT → CI**	**0.031**	**0.031**	**0.014**	**2.167**	**0.031**

Note: Bold path indicates significant indirect effect.

Perceived usefulness was shown to be the most important determinant of continuance intention, followed by perceived computer self-efficacy and satisfaction, while confirmation caused the most variation in satisfaction followed by perceived usefulness. Confirmation was also found to be causing most variation in perceived usefulness followed by perceived computer self-efficacy. As a result, MOOC characteristics of perceived usefulness has a larger influence on continuance intention than any other construct. Between confirmation and continuance intention, as well as confirmation and satisfaction, and between self-efficacy and continuance intention, perceived usefulness served as an important mediating variable. Learners will continue to use MOOCs and be satisfied if they deliver necessary functionality and confirm their initial expectations. Learners will be more satisfied with MOOCs and will be more likely to continue using them if the post-use usefulness is higher.

The ECM model was extended by adding MOOC characteristics (perceived openness and perceived reputation) and other learner-specific features (perceived enjoyment and self-efficacy). Perceived openness had a non-significant impact on satisfaction. This result is agreeable with that of Chen et al., (2018) and Alraimi et al., (2015). The second MOOC feature, perceived reputation also demonstrated no significant impact on satisfaction which is consistent with Khurana et al., (2019). However, there are very limited studies to confirm the relationship between perceived openness and perceived reputation on satisfaction. The results confirmed that perceived enjoyment is not a significant indicator of satisfaction, and this finding is consistent with that of Kim (2011). Nevertheless, the findings support the significant impact of confirmation on perceived enjoyment.

The results of specific indirect effect (refer to Table 6) suggest the impact of confirmation on continuance intention through satisfaction and also self-efficacy on continuance intention. Self-efficacy is found to be a significant indicator of continuance intention and perceived usefulness and this result is consistent with that of Al-Rahmi et al., (2020); Sayaf et al., (2021); Al-Adwan (2020); John (2013). It has to be noted that self-efficacy also demonstrates a significant indirect effect on continuance intention through perceived usefulness. Because MOOCs are accessed through a web browser with a variety of capabilities and functions, computer self-efficacy promotes perceived usefulness, which influences continuance intention. It can be found that the three rejected hypotheses are related to the perception of users about perceived reputation, perceived openness, and perceived enjoyment which indicates that these factors do not influence the level of satisfaction achieved from MOOC. Since learners picked diverse courses with different assignments, perceived openness does not affect satisfaction. As a result, there are no significant connections between these variables. Further, confirmation has a significant indirect effect on continuance intention through satisfaction and perceived usefulness and on satisfaction through perceived usefulness besides its significant direct effect. The favourable influence of confirmation following the use of MOOCs may strengthen the beneficial effect of satisfaction and perceived usefulness on continuance intention.

The findings suggest that perceived usefulness, perceived computer self-efficacy and satisfaction have direct effect on continuance intention; and confirmation, perceived usefulness and perceived computer self-efficacy have indirect effect on continuance intention to use MOOC. The results also indicate that perceived usefulness and confirmation influence satisfaction and also confirmation affects perceived enjoyment. As stated by Bhattacherjee (2001b), due to the intangible nature of business-to-customer services, the consumers recognized the associated benefits only on the use of service, and thus, the service providers should educate the users or potential customers regarding the potential benefits to enhance its continuance intention. If learners’ first experience contradicts the promises made by MOOC designers or instructors, the resulting unhappiness and unfavourable opinion of post-use utility would make them less likely to return. Furthermore, the more realistic, unbiased, and rewarding first-hand experience learners have, the higher would be the satisfaction with learning as well as the intention to continue. As a result, MOOC creators/instructors should not only inform new learners on the benefits of the courses but also educate current learners on how to use them efficiently to optimize confirmation and perceived enjoyment.

## Theoretical and managerial implications

Most of the extant research focuses on MOOC adoption or acceptance and not continuance intention. Therefore, this study adds to the literature by attempting to predict the continuous intention to use MOOCs using an improvised model that incorporates ECM and other characteristics associated with learning MOOCs, such as perceived enjoyment, perceived openness, perceived reputation, and computer self-efficacy. Also, this study endorses the role of the ECM in affecting MOOC usage. The model’s predictive value is quite high (63.9%), implying that the theoretical model has good explanatory power in predicting the ongoing desire to use MOOCs. It also adds to the literature by corroborating the positive effect of self-efficacy on ex-post expectation, also known as perceived usefulness, and confirmation of expectations that enhances perceived enjoyment in learning courses. Self-efficacy as demonstrated by the accomplishment of online coursework and other learning activities in a digital environment would strengthen perceived usefulness. Unlike other models, our suggested model emphasizes the importance of confirmation in enhancing the perceived enjoyment, thereby extending the ECM model’s emphasis on the role of confirmation on usefulness and satisfaction. Students would enjoy and continue to enrol in MOOCs if the offerings matched their expectations.

Our findings have certain implications for course developers and administrators. The institutions and universities offering MOOCs should focus on improving confirmation of initial expectations and, as a result enhance perceived enjoyment, satisfaction, and perceived usefulness. Users’ perceived usefulness can be increased if computer self-efficacy is promoted through the design of simpler learning and assessment tasks, course delivery through multimedia, and interactive learning activities. Given the significance of perceived usefulness in driving satisfaction and continuance intention, higher education instructors should concentrate on developing courses that improve learners’ industry-required skill sets and their practical application.

Confirmation of expectations has a significant indirect influence on continuance intention through usefulness and satisfaction. After familiarity and realization of the benefits of MOOCs, students may complete the courses and enroll in newer courses to achieve their academic goals. Therefore, the MOOC administrators and instructors should focus on meeting the learners’ expectations through collaborative learning activities such as scenario-based group activities, space, and place, sub-networks, gamification, ePortfolios, and role-playing. Moreover, satisfaction and higher perceived enjoyment (when the provider exceeds the user expectation) will help the provider retain its users. Therefore, the MOOCs platforms should incorporate entertainment elements in course delivery, for example, video streaming, chat rooms, and online discussion forums in addition to content management. To maximize student pleasure and enjoyment, quizzes, playing-and-learning, and other innovative methods should be used to the greatest extent possible via the internet’s multimedia capabilities.

The findings of this study highlight the importance of confirmation, satisfaction, perceived usefulness, and perceived computer self-efficacy and the insignificant effect of perceived openness and perceived reputation. Therefore, MOOC providers should develop strategies to enhance user experiences and meet their expectations rather than the features of MOOC platforms. Personal and subjective experiences do influence the continuance of decisions to the extent that learners’ experiences confirm prior expectations and meet their learning needs. Perceived usefulness can be increased by providing a high-quality learning experience and curriculum resources, as well as ensuring the comfort and pleasure of the learning process. Satisfaction can be increased by using a student feedback mechanism that reflects the actual learning state (Mangaroska & Giannakos, 2019).

MOOC instructors and developers should place a greater emphasis on the benefits of the many courses offered to distinguish them from other rivals. Quality of the website and course contents, ease of locating educational materials, and variety of supporting learning resources (hyperlinks, assignment tools, exams, seminars, teaching materials, curriculum, discussion boards, and course requirements) would improve confirmation of expectations and thereby satisfaction and course usefulness. Certain auxiliary learning tools can be incorporated into the applications to provide a pleasant user experience. Providing materials that aid the process of learning and fulfil the course objectives as well as the needs of learners would contribute to improving satisfaction.

## Limitations and future scope of study

The study is limited to a smaller sample size and few institutes in Karnataka. Replicating this research model with a larger and geographically diverse sample size may give new insights into the relationship between the variables in the study. The results of this study cannot be generalized to a larger population as the study is based on convenience sampling. Future researchers may apply a random sampling method to verify if the results are consistent and if they can be replicated in a larger population. What constitutes perceived openness is questionable. Also the perceived reputation of both platform and course provider needs to be taken into account. Therefore, further inquiry on different aspects of perceived openness and perceived reputation may give some insight into what role they play in satisfaction and continuance intention to use platforms. Because student behaviour is shifting, a longitudinal study of complicated interrelationships could provide more insight into continuance intention for MOOCs. Although the current study focused on satisfaction, future research could include another element of effect namely attitude, personality traits, curiosity, and other factors. While we recognize that factors like MOOC design, access to the internet and high-quality tools and resources, IT skills, learner’s personal circumstances like income, health, and family, as well as risks with impulsive enrolling, may also be influencing students to continue or abandon their education, we were unable to account for these variables due to their complexity. These issues can be addressed in the future studies.

## Conclusion

The increasing popularity and need for e-learning or web-based learning and the increase in the number of courses/platforms offering e-learning/MOOC have made it necessary to understand the intention to use MOOC and the antecedents to the level of satisfaction achieved in using MOOC. This article examines factors that shape continuance intention to utilize MOOC by combining the expectation confirmation model with additional MOOC-related characteristics including perceived enjoyment, perceived openness, and perceived reputation. The empirical results indicate that confirmation affects perceived usefulness, satisfaction, and perceived enjoyment, with perceived usefulness, perceived computer self-efficacy and satisfaction influencing continuation intention directly. Further, perceived computer self-efficacy influences perceived usefulness and perceived usefulness affects satisfaction. However, enjoyment, reputation and openness were not seen to affect satisfaction. These empirical results supports the successful extention of ECM with perceived computer self-efficacy and perceived enjoyment. MOOC platforms ought to give quality courses that are helpful and gratifying to students and enhance their learning while contributing to the development of their skills, knowledge, and values that are valuable to seek out jobs.

## Data Availability

The dataset analysed in current study are not publicly available as the permission for data sharing is not taken from the respondents.
